# Nutritional considerations for glucagon-like peptide-based therapies: An Asian Indian consensus recommendation

**DOI:** 10.1016/j.obpill.2026.100296

**Published:** 2026-07-15

**Authors:** Shashank R. Joshi, Anoop Misra, Ambrish Mithal, Banshi Saboo, Krishna G. Seshadri, Subhankar Chowdhury, Ganapathi Bantwal, Bipin Sethi, Jothydev Kesavadev, Arpan Dev Bhattacharya, Manash P. Baruah, Ajay Budhwar, Priti Thakor, Sachin Shende, Viswanathan Mohan

**Affiliations:** a Department of Endocrinology, Lilavati Hospital, Mumbai, India; b Centre of Excellence for Diabetes, Metabolic Diseases and Endocrinology, Fortis-C-DOC, Delhi, India; c Department of Endocrinology & Diabetes, Max Super Speciality Hospital, Delhi, India; d Department of Diabetology, Dia Care, Diabetes Care & Hormone Clinic, Ahmedabad, India; e Department of Endocrinology and Diabetes, Apollo Hospitals, Chennai, India; f Department of Endocrinology & Diabetes, Manipal Hospital, Kolkata, India; g Department of Endocrinology, St. John's Medical College, Bengaluru, India; h Department of Endocrinology, CARE Hospitals, Hyderabad, India; i Department of Diabetes and Technologies, Jothydev’s Diabetes & Research Center, Trivandrum, India; j Department of Endocrinology & Diabetes, Manipal Hospital, Bengaluru, India; k Department of Endocrinology & Diabetes, Apollo Excelcare Hospital, Guwahati, India; l Department of Internal Medicine, Dr Ajay Budhwar’s Diabetes Clinic, Amritsar, India; m Department of Medical Affairs, Abbott Healthcare Pvt. Ltd., Mumbai, India; n Department of Medical Services, Abbott Healthcare Pvt. Ltd., Mumbai, India; o Department of Diabetology, Madras Diabetes Research Foundation (ICMR- Collaborating Centre of Excellence), Chennai, India; p Department of Diabetology, Dr. Mohan's Diabetes Specialities Centre (IDF Centre of Excellence in Diabetes Care), Chennai, India

**Keywords:** Diabetes specific nutrition formula, Dual glucose-dependent insulinotropic polypeptide (GIP) and glucagon-like peptide (GLP)-1 receptor agonist, Glucagon-like peptide-1 receptor agonists, Nutrition, Obesity, Type 2 diabetes mellitus

## Abstract

**Background:**

Glucagon-like peptide-1 receptor agonists (GLP-1 RAs) and dual glucose-dependent insulinotropic polypeptide (GIP) and GLP-1 receptor agonists, collectively called GLP-based therapies (GBTs), cause gastrointestinal adverse effects and alter dietary intake, leading to nutritional deficiencies and loss of muscle mass and bone density. Therefore, GBTs’ use necessitates structured nutritional support, particularly among Asian Indians, given their diverse dietary patterns and high prevalence of sarcopenia. This consensus provides expert-driven, practical recommendations on key nutritional priorities to support the appropriate GBTs’ use in individuals with type 2 diabetes mellitus and/or obesity.

**Methods:**

This was a consensus-based study using a modified Delphi method with expert panel inputs informed by a literature review. The literature review was conducted before developing statements on challenges experienced by individuals on GBTs and strategies to mitigate micro- and macronutrient deficiencies to guide optimal GBTs’ use. A total of 13 domain experts (endocrinologists and diabetologists) across India were included in this process.

**Results:**

Among 44 statements (11 sub-statements), 43 received high or moderate consensus, emphasizing the importance of early nutritional assessment and monitoring metabolic outcomes, with a tailored, balanced diet comprising complex carbohydrates, optimal high-quality protein, good-quality fat, micronutrients, and adequate hydration. The panel highlighted calorie-controlled meal replacement, including diabetes-specific nutritional (DSN) formulations, together with sustainable lifestyle practices and resistance exercise to support long-term metabolic health outcomes. After the discontinuation of GBTs, sustained nutritional support may help prevent metabolic rebound.

**Conclusion:**

There is a need for structured nutritional support for individuals on GBTs, especially for Asian Indians, considering their dietary pattern and increased risk of sarcopenia. Implementing culturally driven nutritional recommendations, including DSN formula as an adjunct along with lifestyle modifications, can support optimizing long-term patient-centric outcomes. The effectiveness and safety of GBTs, along with newly emerging nutrient-stimulating hormone (NuSH) based therapies, should be evaluated in long-term follow-up studies.

## Graphical abstract

**Figure:**
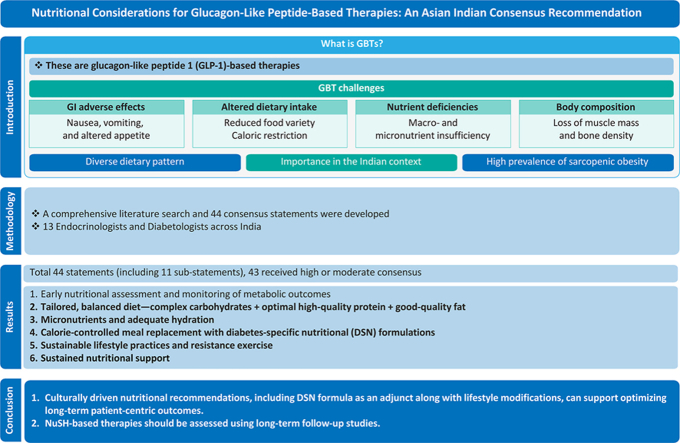

## 1 Introduction

Glucagon-like peptide-1 receptor agonists (GLP-1 RAs) and dual glucose-dependent insulinotropic polypeptide (GIP) and GLP-1 receptor agonists are Food and Drug Administration (FDA)-approved medications recommended as adjuvants to diet and exercise for the management of type 2 diabetes (T2D) mellitus and obesity [1]. In 2026, the World Health Organization (WHO) released its first evidence-informed recommendations on the use of GLP-1 RAs and GIP/GLP-1 dual agonists, collectively called GLP-based therapies (GBTs), for the management of obesity [2]. The American Diabetes Association (ADA) also advocates these medicines for individuals living with T2D and obesity to mitigate the risks of kidney and cardiovascular diseases [3]. Clinical trials such as LEAD, STEP, SURPASS, SCALE, SURMOUNT, and SELECT have also demonstrated clinically significant weight loss and reductions in cardiovascular disease morbidity and mortality among individuals on GBTs [4,5]. GBTs mimic the incretin hormone GLP-1 and act through central and peripheral pathways to improve glucose-dependent insulin secretion, reduce appetite and hunger, delay gastric emptying, and maintain satiety and blood glucose levels [1,6]. They activate hypothalamic receptors in the dorsomedial hypothalamus, thereby enhancing pre-ingestive satiation. Furthermore, they reduce the activation of the reward circuit in the brain (insula and orbitofrontal cortex), thereby reducing hedonic response to high-calorie and palatable food [6]. Through this neuroendocrine mechanism of action, GBTs shift the paradigm for obesity management from a calorie-centric approach to neuroendocrine regulation [7].

Despite these benefits, GBTs raise important clinical concerns such as gastrointestinal side effects (nausea, vomiting, diarrhea, and constipation), risk of sarcopenia due to significant loss of muscle quantity and function, and nutritional inadequacies. Table 1, Table 2 present the mechanistic effects of GBTs and India-specific dietary patterns and nutritional vulnerabilities.

**Table 1:** Established evidence and mechanistic effects of GLP-based therapies.

1. **Reduced calorie intake** Due to a 16%–39% reduction in overall caloric intake, altered food choices may predispose individuals to macronutrient and micronutrient inadequacies and compromise overall nutritional status [[8], [9], [10]].
2. **Nutritional deficiencies** Clinical evidence indicates an increased prevalence of nutrient deficiencies, with approximately 20% reported within the first year of initiating GBTs [11]. These deficiencies include vitamin D (7.5% at 6 months; 13.6% at 12 months), iron (26%–30% lower ferritin compared to sodium-glucose cotransporter 2 inhibitors), vitamin B_12_ (1.3% at 6 months, 2.6% at 12 months), along with other vitamins and minerals [9,11].
3. **Protein inadequacy and muscle wasting** Dietary assessment reported that approximately 60% of individuals on GBTs consume less calcium and iron than the recommended levels [9]. Inadequate protein, vitamin D, and calcium intake may further contribute to a progressive loss of lean muscle mass, strength, and function [9,[12], [13], [14]].

**Table 2:** India-specific dietary patterns and nutritional vulnerability.

1. Among Indians an increased risk of micronutrient deficiencies and metabolic disorders are observed due to high consumption of refined/ultra-processed calorie-dense food and vegetarian diets [15,16].
2. High prevalence of micronutrient deficiencies including vitamin D (61%), iron (54%), vitamin B_12_ (53%), folic acid (37%), and iodine (17%), largely owing to cereal-based food are observed [17].
3. Indian Council of Medical Research–India Diabetes(ICMR-INDIAB) data demonstrated a predominant consumption of refined cereals (Northeast: 99%, South: 87%, East: 78%), with higher whole-wheat intake in North India (90%) and Central India (70%). Western Indians also consume a high-fat and low-protein diet [18,19].
4. Refined cereals/ultra-processed foods reduce the intake of bioactive compounds (e.g., myo-inositol), bran, fiber, and micronutrients, contributing to impaired metabolic signaling and gastrointestinal symptoms such as bloating and altered bowel habits [18,[20], [21], [22]].

Additionally, normal weight obesity (NWO), a commonly observed phenotype among Asian Indians, characterized by excessive body fat and normal body mass index (BMI), is associated with a higher risk of sarcopenia among males (22 times) and females (25 times) compared to individuals with normal weight without obesity (NWNO) [23]. The high prevalence (14.2%–39.2%) of sarcopenia has been reported in Indian adults with malnutrition/obesity, as malnutrition/obesity is one of its primary causes [24]. This risk is further increased by high-carbohydrate, low-protein dietary patterns, physical inactivity, and T2D [25]. In addition to these pre-existing nutritional inadequacies stemming from diverse dietary patterns in India, GBTs further raise concerns due to appetite suppression, reduced food cravings, binge eating, emotional and uncontrolled eating, and gastrointestinal side effects. Although a decrease in dietary intake is partially responsible for weight loss, it causes inadequate nutrient intake, leading to micronutrient deficiencies [26].

Hence, nutritional guidelines are of paramount importance for individuals on GBTs. Although there exist global recommendations on nutritional priorities for individuals on GBTs, there is a need for culturally oriented Asian Indian-specific recommendations, given India’s diverse geography and varied, culturally influenced local dietary and food patterns. Published literature also supports the use of transcultural diabetes nutrition algorithms (tDNA), which tailor nutritional guidance to regional dietary patterns and cultural practices, improving adherence and clinical outcomes [27,28]. Therefore, Asian Indian-specific recommendations that integrate nutritional priorities with GBTs to improve patient-centric clinical outcomes are warranted. The goal of this consensus, therefore, was to establish domain-expert-driven, practical recommendations on key nutritional priorities to support the appropriate use of GBTs among Indian adults with T2D and/or obesity.

## 2 Methodology

**Fig. 1:**
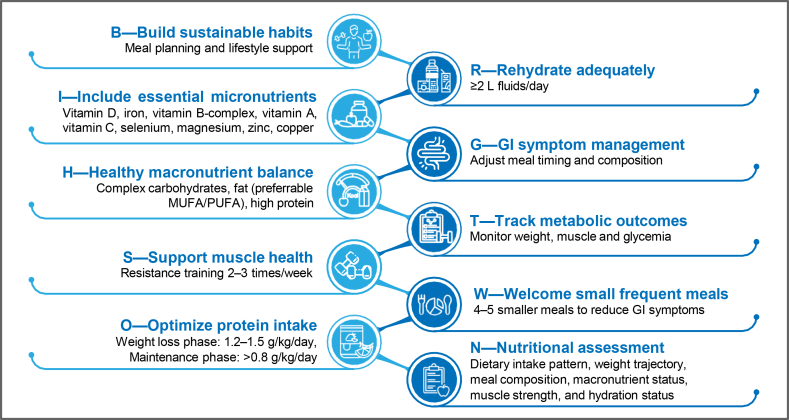
Flowchart summarizing the Delphi process, including the number of statements developed and the consensus achieved.

A modified Delphi technique supported by a comprehensive literature review and expert opinions was used in this study. To ensure transparency and clarity in the process, the ACcurate COnsensus Reporting Document (ACCORD) checklist was used as reporting guidelines for this study consensus [29]. Fig. 1 represents an overview of the consensus workflow. A domain-expert panel of 13 diabetologists and endocrinologists, based on their professional qualifications and extensive clinical experience, was convened across India to ensure nationwide representation across diverse clinical settings and practice contexts. Email invites were sent to the panelists requesting their participation in this consensus process. Participation was voluntary, and informed consent was obtained from each participant prior to the study. One of the panel members acted as a moderator to oversee and guide the consensus process. All panel members were informed of the objectives, methodology, and intended use of the outcomes gathered through this consensus process. All the responses were anonymized to ensure confidentiality.

A detailed literature review was conducted in PubMed/Medline, Google Scholar, and local health registries to understand the challenges reported by individuals on GBTs, interpret nutritional strategies to address macro- and micronutrient deficiencies, and formulate guidelines to incorporate the diabetes-specific formula (DSN) as a standard of care for individuals on GBTs. Systematic reviews, meta-analyses, randomized controlled trials, cohort studies, and previously published consensus guidelines with clearly defined methodology and outcome measures were included for the development of the statements. These statements were further refined based on the moderator’s feedback. The strength and quality of evidence Supplementary Tables 1 and 2 were assessed using the Oxford Centre for Evidence-Based Medicine Level of Evidence (2011) [30]. The consensus process involved two rounds of online voting and one round of discussion among the expert panel members. In the first round, the developed statements were disseminated to all expert panel members, who cast anonymous votes using a 5-point Likert scale Supplementary Table 3 [31]. Following this, a physical steering committee meeting (SCM) was held, and the statements that achieved low and moderate consensus in round 1 were discussed and reframed according to the expert panel’s suggestions. After the SCM, a final round of anonymous voting was done to finalize the statements.

## 3 Results

A total of 44 statements (including 11 sub-statements) were developed after a comprehensive literature review and disseminated for voting. After the first round of voting, 34 statements achieved high consensus, 7 moderate consensus, and 3 low consensus. Nine statements that received low- and moderate-level consensus were deliberated upon and reframed to align with the expert panel’s suggestions and underwent a second round of voting. One of the statements achieving a low consensus was removed after discussion. After the second round of voting, 42 statements received high consensus, and one received moderate consensus Fig. 1.

## 4 Recommendations for Asian Indians

GBTs are widely prescribed for the management of T2D and obesity. However, the associated gastrointestinal side effects and alterations in dietary intake may cause nutritional deficiencies and significant loss of muscle mass and function, and bone density. Understanding the metabolic and nutritional consequences of GBTs is essential for implementing appropriate mitigating strategies.

### 4.1 Understanding the challenges faced by individuals on GBTs [Statements 1–9]

**Fig. 2:**
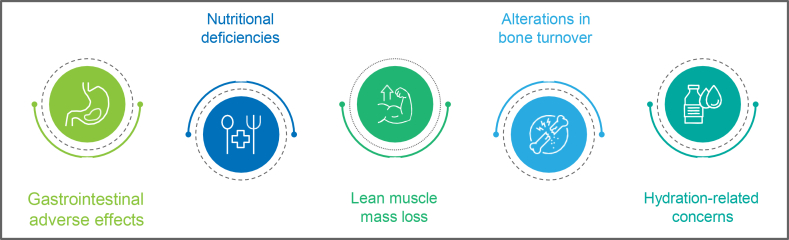
Pentad of challenges in individuals on GBTs GBT: GLP-based therapy.

The results of the modified Delphi consensus indicated high consensus (77%–100%) on nine statements regarding the challenges experienced by individuals on GBTs Table 3. Experts highlighted the pentad of challenges commonly reported by individuals on GBTs Fig. 2.

**Table 3:** Common challenges experienced by individuals on GBTs.

Statement no.	Statement	Voting Score (%)	Level of Evidence
1	Individuals on GBT commonly experience gastrointestinal side effects (nausea, vomiting, constipation) that impact dietary adherence.	100%	1
2	Reduced appetite and caloric intake during GBT can lead to macro- and micronutrient deficiencies if not monitored.	92%	2
3	Rapid weight loss associated with GBT may increase the risk of lean muscle mass loss and compromise bone health.	85%	1
4	Individuals often lack structured nutrition guidance, leading to inconsistent dietary patterns and suboptimal outcomes.	92%	1
5	Poor nutritional management during GBT may compromise achieved metabolic benefits, such as improved glycemic control, cardiometabolic risk profiles, and long-term weight maintenance.	85%	1
6	Inadequate macronutrient intake during GBT may accelerate sarcopenia in older adults.	77%	3
7	Failure to address micronutrient needs can result in fatigue, anemia, and impaired immunity, reducing quality of life.	100%	2
8	Lack of hydration strategies may increase risk of constipation and kidney stress.	77%	4
9	Individuals who discontinue the GBT may regain approximately two-thirds of their prior weight loss within one year due to inadequate lifestyle and nutritional support.	85%	1

GBT: GLP-based therapy.

**Fig. 3:**
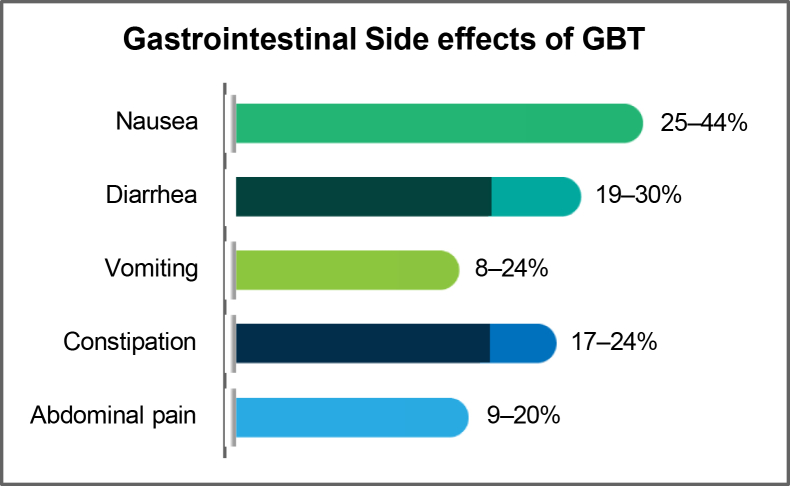
Gastrointestinal side effects experienced by individuals on GBTs GBT: GLP-based therapy.

Gastrointestinal side effects are reported among 40%–70% of individuals on GBTs. These side effects occur irrespective of the half-life of the drugs (short-/long-action) or route of administration (oral/subcutaneous). These side effects are usually mild to moderate and transient, commonly occurring during initiation or up-titration of the dosage, which often decrease with gradual titration or dose reduction. The underlying cause of these side effects may vary, and the side effects may further increase with higher dosage, indicating a dose–response relationship [32]. Fig. 3 depicts the gastrointestinal side effects experienced by individuals on GBTs [[33], [34], [35]].

GBTs decrease intestinal motility and gastric emptying through the central nervous system (vagal pathway) or direct action on central receptors, which leads to these gastrointestinal side effects [36]. These side effects also reduce dietary intake (a 16%–39% reduction in caloric intake has been reported), thereby affecting the nutritional status of individuals on GBTs. Less common side effects include fatigue, hair loss, dyspepsia, gastroesophageal reflux, eructation (belching), dizziness, gastritis, hypoglycemia, gallbladder disease, pancreatitis, acute kidney injury (due to hypovolemia), hypersensitivity reactions, and gastroparesis [35]. Rare side effects such as acute pancreatitis, cholelithiasis, medullary thyroid carcinoma, diabetic retinopathy, and non-arteritic ischemic optic neuropathy have also been reported, although the incidences of such cases are very low [37]. GBTs are relatively newer therapeutic drugs and are still evolving into multifunctional metabolic drugs. Hence, long-term pharmacovigilance studies are warranted, which can provide comprehensive evidence on the clinical consequences, safety, and efficacy of GBTs [38]. Reduced thirst perception and water intake have been reported with GBTs. Lower intake of fruits and vegetables also compromises water intake, as many of these are composed of 90% water. A decrease in water consumption reduces glomerular filtration rate and increases serum creatinine levels, which negatively impacts renal function [39,40]. Sodium excretion also increases with liraglutide, which further impairs body fluid balance [41]. Liraglutide, semaglutide, and lixisenatide are associated with acute kidney injury due to nausea, vomiting, or dehydration in some cases, although this association of GBTs and acute kidney injury has not been reported by larger studies [41].

GBTs also increase the risk of sarcopenia. This is more relevant in the Asian Indian context, as South Asians have lower baseline lean muscle mass relative to body size and a greater propensity for central (visceral) fat accumulation compared to Western populations. Reduced muscle mass, impaired fat utilization, and increased visceral-to-peripheral adiposity further increase the risk of sarcopenic obesity [[42], [43], [44]]. In India, the prevalence of primary sarcopenia is high (14.2%–39.2%) among adult populations, and 1/4 of these adults report having sarcopenic obesity [24]. Further, an advanced age and a positive diagnosis of diabetes predispose individuals to a higher risk of lean muscle mass loss even without GBTs [10]. Older adults (>65 years) may be at an increased risk of sarcopenia while losing weight due to reduced lean muscle mass as well as anabolic resistance, which impairs protein synthesis in response to resistance training or dietary protein intake. After the discontinuation of GBTs, the risk may be further aggravated as the rapid weight regain may be likely due to fat gain. Adding to micronutrient deficiencies owing to reduced food intake, the muscle mass loss leads to an increased risk of frailty among older individuals [45].

The loss of lean muscle mass among individuals on GBTs may also be due to “yo–yo” dieting, lack of physical activity, and poor dietary intake (protein-deficient). Jio et al. (2024) reported that the loss of lean body mass was higher in individuals receiving GBTs than in controls [46]. Karakasis et al. (2025) also found that GBTs significantly reduced total body weight (mean difference [MD] −3.55 kg, 95% confidence interval [CI] [−4.81, −2.29]), fat mass (MD −2.95 kg, 95% CI [−4.11, −1.79]), and lean mass (MD −0.86 kg, 95% CI [−1.30, −0.42]), with lean mass loss comprising approximately 25% of the total weight loss [47]. In a retrospective cohort study by Ren et al. (2025), the semaglutide-treated group reported significantly lower BMI, appendicular skeletal muscle mass index, gait speed, and handgrip strength [48]. The SEMALEAN study reported a significant impact of semaglutide on weight loss, body fat, lean muscle mass, and metabolic efficiency. Lean muscle mass decreased by 3 kg over 7 months, then stabilized between 7 and 12 months; however, handgrip strength increased significantly (+4.5 kg) [49]. However, the loss of lean muscle mass is not solely related to GBTs but also to the population, drug therapy given, and comorbid conditions among individuals. Thus, factors such as the individual’s age and comorbid conditions should be considered before initiating GBTs [50]. Joshi et al. (2025) also highlighted this paradox in the SEMALEAN study, in which lean mass reduction with semaglutide was accompanied by improved muscle strength, enhanced energy efficiency, altered muscle physiology, and modulation of resting energy expenditure, possibly reflecting a shift toward better muscle quality and neuromuscular efficiency. These effects are more pronounced among women than men. However, future studies focusing on mechanistic biomarkers and lifestyle-based interventions stratified based on gender to clarify this functional–metabolic dissociation and enhance the interpretability of the study were suggested [51].

A summary of clinical evidence on the impact of GBTs on body composition is mentioned in Table 4.

**Table 4:** Summary of clinical evidence on body composition changes associated with GBTs.

Trial	Drug/dose	N (on active)	Weeks/months	Weight loss	Lean muscle loss	Fat mass loss
**STEP-1 DEXA sub study** [52]	Semaglutide 2.4 mg	140 (95)	68 weeks	−15%	−9.7%	−19.3%
**Caballero-Mateos *et al.*** [53]	Semaglutide 0.25–1 mg	117	12 months	−11.2 kg	−2.2 kg	−9.7 kg
**SEMALEAN study** [49]	Semaglutide 2.4 mg	106	12 months	−12.7%	−3 kg	−18.9%
**SUSTAIN 8 subset** **DXA** [54]	Semaglutide 1 mg	114 (53)	52 weeks	−5.7 kg	−2.3 kg	−3.4 kg
**Blundell *et al.*** [55]	Semaglutide 1 mg	30	12 weeks	−5.0 kg	−1.1 kg	−3.5 kg
**SURMOUNT 1 DXA sub study** [56]	Tirzepatide 15 mg	160 (124)	72 weeks	−21.3%	−10.9%	−33.9%

In addition to GBTs’ impact on lean muscle mass loss, their impact on bone health has also been reported. Hansen et al. (2024) reported a higher level of bone resorption marker in the semaglutide group than in the placebo group (estimated treatment difference 166.4 ng/L (95% CI 25.5–307.3; p = 0.021) [57]. In February 2026, Liu et al. reported that treatment with semaglutide or tirzepatide was associated with a greater annualized decline in total hip bone mineral density in individuals without diabetes mellitus. However, among individuals with diabetes mellitus, total hip bone loss was comparable between those receiving GBTs and the control group. These findings suggest that the impact of GBTs on bone health may vary according to diabetes status, with treatment-related weight loss potentially contributing to greater bone loss in individuals without diabetes mellitus [58]. In contrast, Norena JA et al. (2025) reported a 26% lower risk of fracture in the semaglutide group compared with sleeve gastrectomy among women with obesity [59]. In addition, Anastasilakis A et al. (2024) conducted a critical appraisal of anti-obesity medications and reported that fracture risk is not increased with GBTs at clinically relevant dose levels, and a few preclinical studies also reported positive effects on bone [60]. Thus, future prospective studies analyzing bone mineral density and bone resorption markers are suggested to validate the results and support clinical decision-making.

Along with these side effects, weight regain post-discontinuation of GBTs is also a concern. Wilding et al. (2022) reported findings from a randomized, placebo-controlled trial conducted at 129 sites across 16 countries, in which participants regained two-thirds of their prior body weight, and cardiometabolic parameters returned nearly to baseline levels approximately one year after completion of semaglutide therapy [61]. Quarenghi et al. (2025) and Budini et al. (2025) reported 11.6%–75.3% weight rebound within 6–12 months [62,63]. This weight regain ranged from 1.50 kg to 5.63 kg as reported in the published literature [[64], [65], [66]]. Table 5 presents the summary of metabolic rebound among individuals post-discontinuation of GBTs.

**Table 5:** Summary of clinical evidence of metabolic rebound after the discontinuation of GBTs.

Study/trial	Drug/dose	N (on active)	Weeks/months	Weight rebound	Metabolic rebound
STEP-1 Extension [61]	Semaglutide 2.4 mg	1961	52 weeks	+11.6	At 120 weeks (mean change) HbA_1c_ rebound ≈ +0.4%, SBP: +10 mmHg, DBP: +4 mmHg, Total cholesterol: ∼+7 mg/dL, Triglycerides: ∼+27 mg/dL
STEP-4 withdrawal trial [67]	Semaglutide 2.4 mg	803	48 weeks	+6.9%	At 68 weeks (mean change) HbA_1c_: 0.1% SBP: +4.4 mmHg Total cholesterol: +11% Triglycerides: +15% Waist circumference: +3.3 cm
SURMOUNT-4 *post hoc* analysis [68]	Tirzepatide 10 mg and 15 mg	308	52 weeks (week 36–88)	<25%: 3.2% 25%–50%: 11.7% 50%–<75%: 18.5% >75%: 22.9%	At week 88 (mean change) HbA_1c_: +0.2%, SBP: +8.7 mmHg, DBP: +3.2 mmHg, Triglycerides: +16.1 mg/dL

DBP: Diastolic blood pressure; GBT: GLP-Based Therapy; HBA_1c_: Glycated hemoglobin; SBP: Systolic blood pressure.

### 4.2 Nutritional strategies to address macro-/micronutrient deficiencies [Statements 10‬–20]

The results of second round of voting indicated high consensus (85%–100%) across all statements on nutritional strategies to address macro-/micronutrient deficiencies among individuals on GBTs Table 6.

**Table 6:** Nutritional strategies to address macro- and micronutrient deficiencies.

Statement no.	Statement	Voting Score (%)	Level of Evidence
10	Individualized nutrition plans are essential to maximize GBT benefits and minimize adverse effects.	100%	3
11	Adequate and good-quality protein intake (20–30% of energy) should be prioritized to preserve lean mass during weight loss.	92%	1
12	Nutrition counseling should emphasize small, frequent meals and good-quality fat, high-fiber foods to manage GI symptoms.	85%	2
13	Ongoing behavioral support and dietary adherence strategies are critical for long-term success.	100%	1
14	Micronutrient monitoring may be integrated into GBT protocols, and supplementation to be considered for individuals with reduced intake or documented deficiencies.	92%	2
15	Adequate hydration (fluid intake of >2 L per day of water or non-sugary drinks) should be part of patient education to prevent dehydration.	84%	5
16	Combining nutrition therapy with resistance exercise enhances metabolic outcomes and prevents muscle loss.	100%	5
17	Mixed fiber interventions with adequate intakes of both soluble fiber (e.g. oat fiber) and insoluble fiber (e.g. whole wheat) could be recommended to manage GI symptoms without worsening bloating.	92%	2
18	Meal timing adjustments (e.g., eating slowly and avoiding large evening meals) can improve tolerance and reduce nausea.	100%	5
19	Patient education on nutrient-dense foods is necessary to ensure adequate micronutrient intake despite reduced appetite.	92%	3
20	Nutritional adequacy monitoring should be done on timely basis alongside GBT monitoring.	100%	4

GBTs: GLP-Based Therapy; GI: Gastrointestinal.

**Fig. 4:**
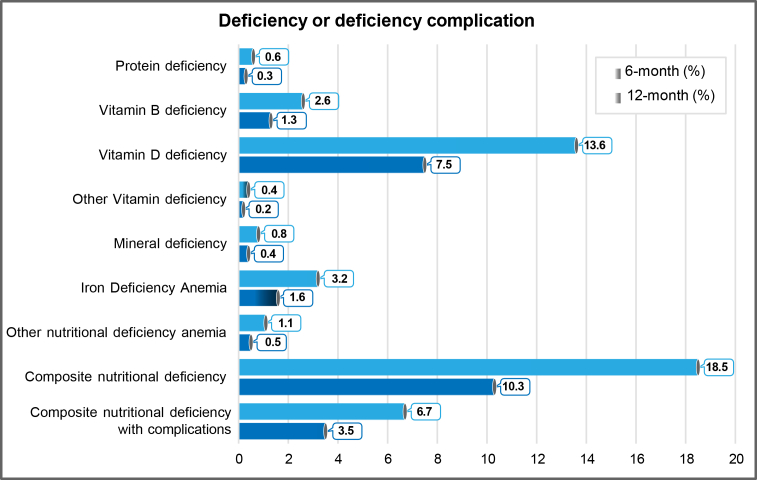
Micronutrient deficiencies among individuals on GBTs [Adapted from Butsch SW et al., (2025)].

Diet is a crucial factor in maintaining glycemic control and managing weight, and this awareness is often lacking among individuals on GBTs [10]. Scott et al. (2025) reported nutritional deficiencies in 12.7% and 22.4% of individuals at 6 and 12 months after initiating GBTs, respectively. Fig. 4 depicts micronutrient deficiencies reported among individuals on GBTs [11].

The near doubling of the prevalence of nutritional deficiencies from 6 to 12 months among individuals on GBTs underscores the importance of proactive screening, diagnosis, and periodic monitoring, with nutritional counseling and tailored interventions [11]. ICMR recommends 0.83 g/kg/day protein intake for healthy Indian adults [69]. The European Society for Clinical Nutrition and Metabolism recommends a protein intake of 1.0 g/kg body weight, with daily moderate dietary restriction to prevent/improve complications associated with obesity. Previous studies have reported that 1.2–1.5 g/kg daily (up to 1.6 g/kg body weight in individuals with normal kidney function) protein intake preserves lean mass and improves body composition during weight loss in young, middle-aged, and older adults, compared with normal protein intake (0.8 g/kg daily) [35,70,71]. Kim et al. (2016) reported that retention of lean mass and loss of fat mass are greater when high-protein diets are consumed by older adults [72]. In addition to protein intake, unsaturated fats and high fiber are also recommended for individuals on GBTs, as these nutrients help modulate gastric emptying and improve gastrointestinal tolerance.

### 4.3 Recommendations to address macro- and micronutrient deficiencies among individuals on GBTs [Statements 21–34]

The results of second round of voting showed high (77%–100%) and moderate consensus (69%) on 22 and 1 statements, respectively. Table 7.

**Table 7:** Defining protocol to incorporate DSN as a standard of care for individuals on GBTs.

Statement no.	Statement	Voting Score (%)	Level of Evidence
21	A comprehensive baseline nutritional assessment is essential before initiating GBT.	92%	1
22	A comprehensive baseline physical assessment in individuals at high risk of losing muscle mass (e.g., individuals with sarcopenic obesity), including muscle strength, should be evaluated using functional tests (e.g., hand grip strength and chair stand test).	100%	4
23	Nutrition counseling should include strategies to maintain adherence and prevent rebound weight gain.	100%	1
24a	Most critical nutritional consideration for individuals starting GBT is to manage lean muscle mass and bone health.	100%	1
24b	Most critical nutritional consideration for individuals starting GBT is to manage nutrient adequacy.	100%	3
25	While addressing nutrition, the requirements must not be skewed to a single nutrient profile, e.g., only protein supplementation or only fiber supplementation.	100%	3
26	Macronutrient supplementation considered should include good-quality protein (PDCAAS 1), good-quality fat (with predominantly MUFA), and complex good carbohydrate (resistant maltodextrin, maltitol, isomaltulose) in desired ratios (Protein 15%–25%, Carbohydrate 45%–60%, Fat 15%–30%).	85%	2
27	Micronutrient supplementation considered could include micronutrients like Vitamin D, Mg, Vitamins (B complex, C, E), Zn, Cr, Se, along with calcium	100%	1
28	Ideal formulation for supplementing macro and micronutrients should have the following characteristics: a. nutrient-dense meal at lower volume	100%	1
	b. Good-quality protein (PDCAAS 1) at 15%–25%	92%	2
	c. Good-quality fat (with predominantly MUFA) at 15%–30%	85%	2
	d. complex carbohydrate (isomaltulose, maltitol, resistant maltodextrin) at 45%–60%	92%	2
	e. Micronutrients like Vitamin D, Mg, Vitamins (B complex, C, E), Zn, Cr, Se along with calcium.	92%	1
	f. Address sensory challenge (altered taste)	85%	2
	g. Helps reduce cardiometabolic risk	85%	1
	h. Helps reduce visceral adiposity	77%	1
	i helps reduce fat mass	77%	1
29	Diabetes-specific nutrition formulas used as partial meal replacements (i.e., partially replacing 1–2 meals/day as part of a calorie-restricted intervention) can reduce body weight, waist circumference, and blood pressure, and improve glycemic control.	77%	1
30	Appropriate nutritional recommendations are needed alongside GBT including Diabetes-specific nutrition formulas. Such recommendations are aimed at preserving lean muscle mass, supporting weight reduction, minimizing adverse events and optimizing glycemic control	92%	1
31	Proper nutritional guidance is must after discontinuation of GBT. Post discontinuation/de-escalation of therapy, appropriate dietary intervention/Diabetes-specific Nutrition formulas could be continued for the long term to prevent metabolic rebound.	92%	1
32	Appropriate nutrition plan including Diabetic-Specific Nutrition formula with right quality and quantity of macro as well as micronutrients could be added from initiation and may be continued throughout the duration along with GLP based therapy to maximize weight loss and metabolic benefits.	100%	1
33	Ideal nutritional formulation with GLP-1 based therapy are evolving. They should include optimal ingredients of macro and micro-nutrients. In the Indian context, reduction of carbohydrates and fats along with enhancement of proteins is recommended. In the micro nutritional recommendation, many ingredients, for e.g. myo-inositol, chromium may have clinically proven benefits.	77%	1
34	Insulin-sensitizing nutrients like chromium, zinc, selenium, myo-inositol may be included as part of a nutritional formula. Ingredients like myo-inositol may have beneficial effects at clinically proven levels.	69%	1

Cr: Chromium; GBT: GLP-Based Therapy; GLP-1: Glucagon-like peptide-1; MUFA: Monounsaturated fatty acids; PDCAAS: Protein Digestibility Corrected Amino Acid Score; PUFA: Polyunsaturated fatty acids; Mg: Magnesium; Se: Selenium; Zn: Zinc.

Nutritional recommendations should lead to positive health outcomes with weight loss [73]. A comprehensive nutritional assessment at baseline and regular monitoring are recommended to ensure safe and effective patient-centric outcomes of GBTs. The assessment should include the patient’s medical history, including conditions relevant to GBTs, such as gastrointestinal disorders, eating disorders, sarcopenia, renal impairment, weight trajectory, and goals for weight reduction [35]. Individuals at high risk of sarcopenia should be screened for skeletal muscle function using validated questionnaires (e.g., hand grip strength, assistance with walking, rising from a chair, climbing stairs, and falls [SARC-F]), followed by screening for markers such as high BMI and waist circumference at initiation of GBTs. Monitoring with dual-energy X-ray absorptiometry and bioelectrical impedance analysis can be done to measure skeletal muscle mass based on affordability and availability among older adults or cases of sarcopenic obesity [24]. This early assessment acts as a benchmark for changes in body composition, nutritional inadequacies, and the risk of sarcopenia during the regular monitoring phase, enabling timely management with targeted nutritional intervention and supervised resistance exercise [74]. Nutritional counseling also plays a critical role in addressing nutritional inadequacies, preserving lean muscle mass, and promoting sustainable, healthy dietary habits. Macronutrient supplementation should be a nutrient-dense, low-volume meal containing high-quality protein [15%–25%, protein digestibility corrected amino acid score (PDCAAS) 1], good-quality fat [monounsaturated fatty acid (MUFA-based fats, 15%–30%], and complex carbohydrates (45%–60%), along with essential micronutrients (e.g., vitamin D, B complex, C, E, magnesium, zinc, chromium, selenium, and calcium) [[75], [76], [77]].

These supplements support reductions in cardiometabolic risk, visceral adiposity, and fat mass while maintaining palatability [78]. Consistent with Research Society for the Study of Diabetes in India (RSSDI) and ICMR guidelines, the Dietary Reference Intake (DRI) also advocates MyPlate proportions of complex carbohydrates (45%–65%) such as whole grains, millets, and pulses; fats (20%–35%) predominantly from plant-based oils and nuts; and proteins (10%–35%) such as pulses, dairy (milk, curd, paneer), soy products, eggs, and lean meats, with further tailoring based on metabolic goals and comorbidities [69,78]. American Diabetes Association (ADA) further emphasizes personalized nutrition therapy within these ranges to support clinically meaningful weight reduction (≥5%) and optimize metabolic outcomes [79].

**Fig. 5:**
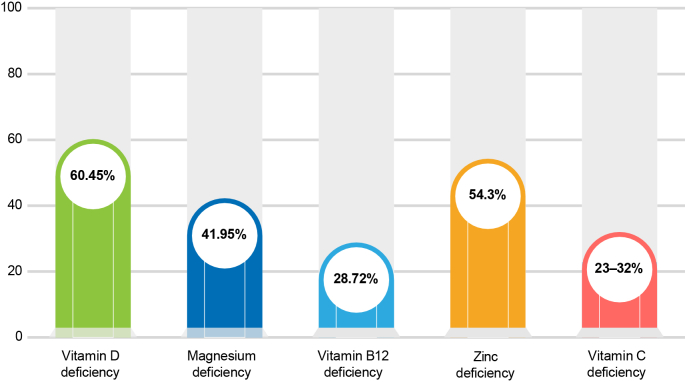
Micronutrient deficiencies among individuals living with T2D.

Micronutrient supplementation should also be considered in individuals with T2D, particularly during treatment with GBTs, as both T2D and GBT usage may predispose individuals to nutritional inadequacies [80]. Evidence indicates that micronutrient deficiencies are common among individuals with T2D, with a pooled prevalence of 45.3% (95% CI 40.35–50.30) for multiple deficiencies, with female predilection (48.62%, 95% CI 42.55–54.70). Fig. 5 represents micronutrient deficiencies among individuals living with T2D [81]. Jayashri *et al* (2018) and Jayashri *et al* (2020) also reported higher prevalence of vitamin B_12_ and vitamin D deficiencies among individuals with T2D, followed by people with prediabetes, compared to normoglycemic individuals [[82], [83], [84], [85]].

Certain micronutrients also exert insulin-sensitizing effects and could be considered as components of nutritional formulations for individuals with impaired glucose metabolism. Fatahi et al. (2025) reported the effects of chromium supplementation in individuals with T2D or insulin resistance and reported significant improvements in insulin resistance indices, such as Homeostatic Model Assessment for Insulin Resistance (HOMA-IR) pooled mean difference (MD) −1.29; 95% CI −1.84 to −0.73; p < 0.001 and fasting blood glucose (pooled MD −13.71 mg/dL; 95% CI −26.29 to −1.12; p = 0.03), suggestive of the beneficial role of chromium in improving insulin sensitivity [86]. Similarly, inositol compounds may improve glucose metabolism through insulin-mimetic mechanisms. Consistent with these findings, Minambres et al. (2019) reported that inositol supplements improve insulin sensitivity, reduce fasting plasma glucose, insulin, and HOMA-IR, independent of weight changes [87]. Collectively, these findings suggest that insulin-sensitizing micronutrients such as chromium, zinc, selenium, and myo-inositol may be considered for inclusion in nutritional formulations. Tey et al. (2024) also reported improvements in HOMA-β, glycated hemoglobin (HbA_1c_) (−0.50% vs. −0.21%), weight (−1.74 kg vs. −0.76 kg), and visceral fat (−6.52% vs. −0.95%) among individuals receiving DSN formula with myo-inositol [88].

Therefore, an ideal nutritional formulation should provide both macro- and micronutrients [81,89]. These supplements should be nutrient-enriched, smaller meals to maintain satiety, dietary adherence, and effective weight management [9,35,73,90]. They should provide a balanced MyPlate composition aligning with diabetes nutrition recommendations [70,78,91]. These formulations should also consider altered taste sensation associated with GBTs to maintain palatability and adherence [92]. Collectively, such nutrient-balanced formulations play a crucial role in maintaining blood glucose levels, reducing cardiometabolic risk factors, and supporting body composition, including reductions in visceral adiposity and fat mass, when incorporated into structured dietary strategies, especially in the Indian population, considering varied dietary patterns [93].

Weight and metabolic rebound following discontinuation of GBTs also pose significant challenges in long-term management. Hence, behavioral strategies, including meal planning, portion control, and reinforcement of healthy eating patterns, can enhance weight maintenance and improve long-term patient-centric treatment outcomes [18,66]. Hence, appropriate nutritional guidance for individuals on GBTs remains standard of care even after treatment discontinuation.

DSN formula with balanced macro- and micronutrients may be considered when individuals are unable to meet their nutritional requirements solely through food [94]. Incorporating DSN formula as a total meal replacement (TMR) or partial meal replacement (PMR) with 1–2 servings/day alongside standard care can significantly improve metabolic outcomes in overweight and obese adults with T2D, including greater reductions in HbA_1c_ (−0.50% vs. −0.21%; p = 0.002), body weight (−1.74 kg vs. −0.76 kg; p < 0.001), and visceral adiposity (−6.52% vs. −0.95%), while also maintaining fat-free mass [88]. Evidence also indicates that formulations enriched in MUFAs and low-glycemic carbohydrates can reduce body weight, waist circumference, and blood pressure while improving glycemic control [94]. Sanz-París et al. (2020) showed that DSN formulas improve postprandial glycemia and metabolic parameters compared with standard nutritional formulas [95]. Similarly, Tey et al. (2024) and Chen et al. (2025) demonstrated that the incorporation of DSN formulas as PMR improved HbA_1c_, body weight, body composition, and cardiometabolic risk markers in adults with T2D [88,96]. Collectively, these findings support the use of DSN formulas as a practical dietary strategy to enhance glycemic management and support weight reduction. The expert panel also suggested incorporating these supplements into dietary management during GBT dose initiation, dose escalation, and maintenance phases.

Another critical factor in facilitating tailored nutritional and lifestyle management is adherence to interventions recommended by trained professionals. A tailored communication strategy, emphasizing the benefits of GBTs and consequences of non-adherence, should be part of initial sessions before GBTs, followed by structured follow-ups at regular intervals. This requires an interdisciplinary approach integrating education and digital innovations [97].

## 5 Practical clinical recommendations for lifestyle management during GBT

### 5.1 Nutritional strategies

**Fig. 6:**
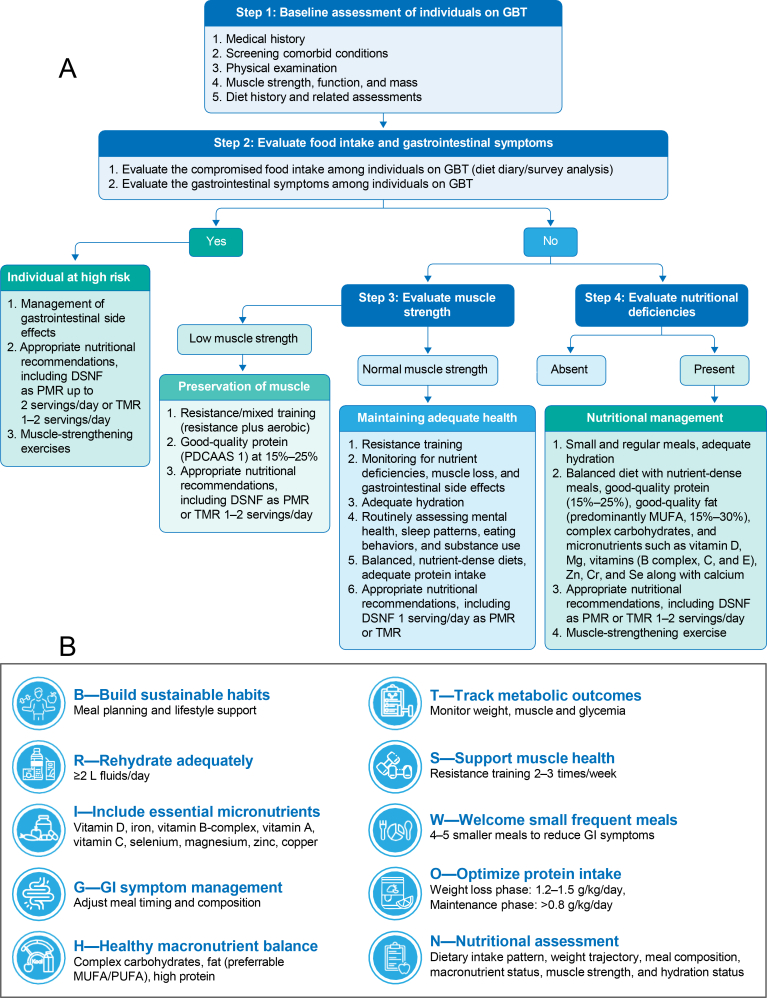
a Practical clinical recommendations for nutritional considerations as an adjunct in individuals on GBTs [26,35,80,98]. DSNF: Diabetes-specific nutrition formula; GBT: GLP-based therapy; TMR: Total meal replacement; PMR: Partial meal replacement. b: Practical clinical algorithm for nutritional support GI: Gastrointestinal; MUFA: Monounsaturated fatty acid; PUFA: Polyunsaturated fatty acid.

Nutritional counseling should be routinely conducted during GBT initiation, dose escalation, and maintenance phases to ensure adequate nutrient intake, improve treatment tolerance, and reduce the risk of metabolic rebound after treatment discontinuation. A few key practical dietary recommendations for physicians and individuals on GBTs are mentioned below. A practical clinical algorithm for nutritional support is presented in Table 8, Fig. 6a and b. The clinical algorithm is intended to guide physicians in monitoring and evaluating individuals on GBTs for nutritional inadequacies and recommend appropriate nutritional strategies.

**Table 8:** Recommendations for nutritional considerations, including DSN supplementation during initiation, dose escalation, and maintenance in people on GBTs.

	Intake Compromised	Muscle Strength	Nutritional Deficiencies	During GBT	
GBT	+	+/−	+/−	PMR/TMR	PMR with 2 meals TMR with 1–2 meals
	–	+	–	PMR/TMR	With 1−2 meals
	–	–	+	PMR/TMR	With 1−2 meals
	–	–	–	PMR/TMR	With 1 meal

GBT: GLP-based therapy; PMR: Partial meal replacement; TMR: Total meal replacement.

Individuals should be thoroughly assessed for medical history (e.g., age at diabetes onset, obesity triggers), weight-reduction goals, relevant comorbidities, physical examination, muscle strength and function, and dietary evaluation before initiating GBTs. During active GBTs, regular monitoring of dietary intake and gastrointestinal symptoms should be conducted to enable early intervention for nutritional inadequacies, reduced muscle strength, and loss of lean body mass. To mitigate these risks, physicians should counsel individuals on maintaining nutritional adequacy through a balanced, nutrient-dense diet, including high-quality protein (15%–25%), predominantly MUFA-based fats (15%–30%), complex carbohydrates, and essential micronutrients (e.g., vitamins D, B-complex, C, and E, magnesium, zinc, chromium, selenium, and calcium). Appropriate nutritional recommendations, including DSN formula (PMR 2 servings per day or TMR 1–2 servings per day), should be recommended to individuals who may be at high risk of nutritional deficiencies due to compromised dietary intake and/or gastrointestinal symptoms. Additionally, structured resistance training or combined (aerobic plus resistance) exercise should be recommended to preserve muscle mass and overall functional health. To ensure adequate muscle strength and dietary intake among individuals without any nutritional deficiencies or muscle strength loss, appropriate nutritional recommendations, including DSN formula (1 serving per day as PMR/TMR), along with resistance training, are recommended. In individuals with low muscle strength or nutritional deficiencies without compromised food intake, appropriate nutritional recommendations, including DSN formula 1–2 servings/day as PMR or TMR, along with muscle-strengthening exercises, are recommended [26,35,80,98]. Table 9 presents nutritional recommendations for physicians managing individuals on GBTs and for individuals receiving GBTs [[75], [76], [77]].

**Table 9:** Nutritional recommendations for physicians and individuals.

Nutritional recommendations for physicians managing individuals on GBTs:	Nutritional recommendations for individuals on GBTs:
1. A comprehensive baseline nutritional assessment is essential before initiating GBTs. 2. Incorporate structured nutritional counseling with GBTs to maximize benefits and help prevent rebound. 3. Ensure adequate good-quality protein intake, such as lean meat, milk, milk products, tofu, etc., by starting progressively (from 0.8 g/kg/day up to 1.2–1.5 g/kg/day or higher) for lean mass preservation and improvement in body composition. 4. Recommend low-glycemic-index carbohydrates such as whole grains, millets, and legumes, and restrict refined carbohydrates. 5. Ensure good-quality fat intake (predominantly MUFA such as rice bran oil, groundnut oil, sesame oil, olive oil, etc.) as 15%–30% of the diet. 6. Advise gradual escalation of dietary fiber intake (target 20–30 g/day) with adequate hydration. 7. Consider DSN as a protocol-driven nutritional intervention, including use as PMR or TMR (1–2 servings/day) when oral intake is inadequate. 8. Advise mixed fiber interventions with adequate intakes of both soluble fiber (e.g., oat fiber) and insoluble fiber (e.g., whole wheat) to manage gastrointestinal symptoms without worsening bloating. 9. Recommend adequate hydration.	1. Eat small, frequent meals (4–5 times/day) instead of large portions. 2. Include good-quality protein (lean meat, milk, milk products, tofu, etc.), good fat (predominantly MUFA such as rice bran oil, groundnut oil, sesame oil, olive oil etc.) and high-fiber foods (fruits, vegetables, oats, whole grains, etc.) to manage gastrointestinal symptoms and preserve lean muscle mass. 3. Stay well hydrated by sipping fluids regularly throughout the day (about 2 L/day), preferably water or unsweetened beverages. 4. Eat slowly, mindfully, and stop when you feel comfortably full. 5. Prefer simple cooking methods such as steaming, boiling, grilling, or light sauteing

### 5.2 Physical activity and resistance training

**Fig. 7:**
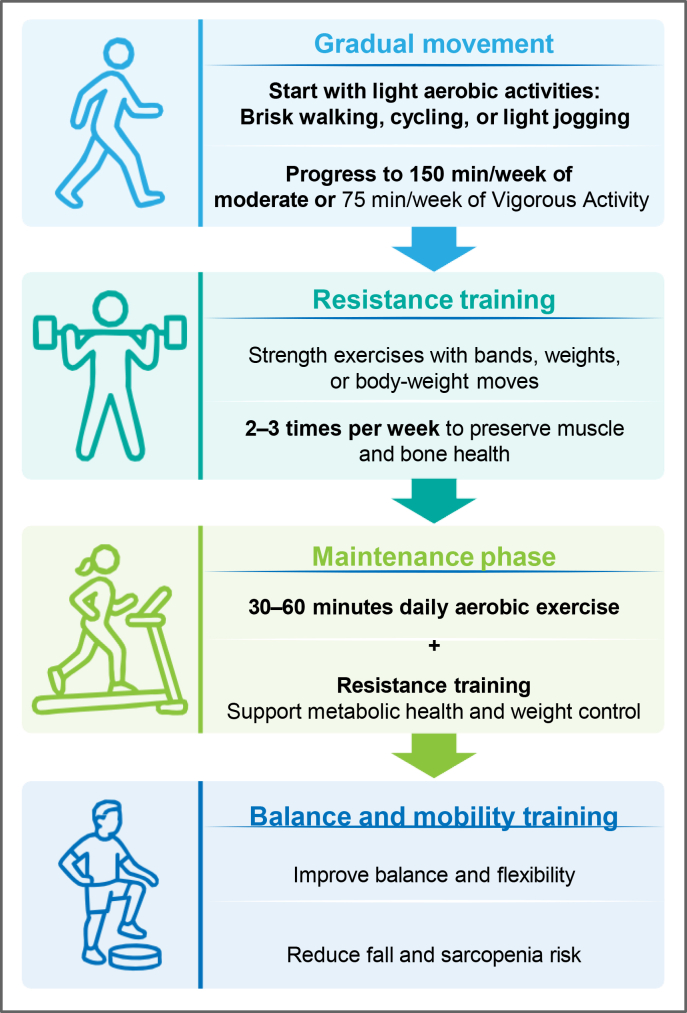
Recommended stepwise physical activity approach.

Regular physical activity is an essential component of care for individuals receiving GBTs, as it helps preserve lean muscle mass, reduce fatigue, and enhance the metabolic benefits of therapy. Exercise should be individualized based on patient fitness, comorbidities, and treatment goals. Evidence suggests that combining pharmacotherapy with structured exercise improves long-term weight maintenance and muscle preservation. A stepwise physical activity approach may be considered Fig. 7.

## 6 Limitations

There are a few limitations that require careful consideration. First, the recommendations are based on heterogeneous evidence and may be affected by the inherent subjective bias of the Delphi process. Second, the rapidly changing therapeutic landscape, along with new long-term data on nutritional interventions and post-GBT outcomes, necessitate future updates to these recommendations.

## 7 Conclusion

Although GBTs can lead to substantial weight loss and are associated with metabolic benefits, several factors may restrict their sustained effectiveness at both individual and population levels. These include gastrointestinal side effects, nutritional inadequacies, reductions in muscle and bone mass, treatment discontinuation due to intolerance to GBTs, and weight/metabolic rebound after discontinuation. Integrating nutritional interventions can help address many of these challenges experienced by individuals on GBTs. Therefore, clinicians prescribing GBTs to people with diabetes and obesity should plan a structured care that includes comprehensive nutritional and lifestyle counseling throughout the treatment journey, before, during, and after the pharmacotherapy. The approach should emphasize balanced dietary practices, regular physical activity with resistance training, and appropriate nutritional recommendations, including DSN formula as an adjunct to address nutritional adequacy and preserve lean muscle mass. This holistic strategy can help achieve long-term patient-centric treatment outcomes and enable clinicians to use GBTs more efficiently. However, future studies assessing the effectiveness and safety of GBTs, practical clinical algorithms, and culturally tailored nutritional support in real-world scenarios are warranted. Nutrient-stimulating hormones (NuSH) are GLP, GIP, glucagon, peptide YY, and amylin-based therapies that have a broader impact on metabolic functions [99]. Although these therapies have shown effectiveness in managing weight-associated comorbidities, long-term studies to assess their safety profile are warranted.

• There is a need for structured nutritional support for individuals on GBTs, especially for Asian Indians, considering their diverse dietary intake and the high risk of sarcopenia.
• Clinicians should recommend an early, structured, culturally tailored, nutritional intervention for individuals on GBTs, including DSN formula, as an adjunct that provides low-glycemic-index carbohydrates, high-quality protein, healthy fats, adequate dietary fiber, and essential micronutrients.
• After the discontinuation of GBTs, sustained nutritional support, including DSN formulas, may help prevent metabolic rebound.

## Author contribution

The concept of the submission was by SRJ, VM, PT, and SS. Data curation, statistical analysis, and funding acquisition were performed by PT and SS. SS participated as an investigator in this study. Methodology, validation, and visualization were contributed by SRJ, VM, PT, and SS. SRJ, VM, PT, and SS wrote the first draft. SRJ, AM, A Mishra, BS, KGS, SC, GB, B Sethi, JK, ADB, MPB, AB, PT, SS, and VM all reviewed, edited, and approved the final submission and publication.

## Ethics approval and consent to participate

Informed consent was sought from the participants of the Delphi consensus. No personal data of the respondents/participants were used during the course of the study.

## Consent for publication

Not applicable.

## Availability of data and materials

Not applicable.

## Declaration of artificial intelligence (AI) and AI-assisted technologies

Not applicable.

## Funding

This consensus was supported by Abbott Nutrition International, India. The funder had no role in the concept or design of the study, data interpretation, manuscript preparation, or the decision to submit the article for publication.

## Declaration of competing interest

The authors declare the following financial interests/personal relationships which may be considered as potential competing interests: **Shashank R. Joshi** reports grants or contracts from Abbott Healthcare Pvt Ltd; consulting fees from Marico, Franco Indian, Zydus Lifesciences, and TwinHealth; and support for attending meetings and/or travel from Abbott, Novo Nordisk, Sanofi, Eli Lilly, Lupin, Alkem, USV, Dr. Reddy’s Laboratories, UNS, and Cipla. **Anoop Misra** reports grants or contracts from Abbott Healthcare Pvt Ltd for the Delphi consensus preparation of this paper, as well as from USV (India), Eli Lilly, Novo Nordisk, Lupin, Cipla, and Zydus; honoraria for educational events from Abbott; and participation on a Data Safety Monitoring Board or Advisory Board. **Ambrish Mithal** reports grants or contracts from Abbott Healthcare Pvt Ltd; honoraria for educational events from Abbott; support for attending meetings and/or travel; and participation on a Data Safety Monitoring Board or Advisory Board. **Banshi Saboo** reports grants or contracts from Abbott Healthcare Pvt Ltd; honoraria for educational events from Abbott; and participation on a Data Safety Monitoring Board or Advisory Board. **Krishna G. Seshadri** reports grants or contracts from Abbott Healthcare Pvt Ltd; honoraria for educational events from Abbott; support for attending meetings and/or travel; and participation on a Data Safety Monitoring Board or Advisory Board. **Subhankar Chowdhury** reports grants or contracts from Abbott Healthcare Pvt Ltd; honoraria for educational events from Abbott; support for attending meetings and/or travel; and participation on a Data Safety Monitoring Board or Advisory Board. **Ganapathi Bantwal** reports grants or contracts from Abbott Healthcare Pvt Ltd; honoraria for educational events from Abbott; support for attending meetings and/or travel; and participation on a Data Safety Monitoring Board or Advisory Board. **Bipin Sethi** reports grants or contracts from Abbott Healthcare Pvt Ltd; honoraria for educational events from Abbott; support for attending meetings and/or travel; and participation on a Data Safety Monitoring Board or Advisory Board. **Jothydev Kesavadev** reports grants or contracts from Abbott Healthcare Pvt Ltd; honoraria for educational events from Abbott; speaker honoraria, consulting/advisory board fees, and funded research support from Abbott, Novo Nordisk, Eli Lilly, Medtronic, Roche, Sanofi, and Boehringer Ingelheim; participation on a Data Safety Monitoring Board or Advisory Board; an unpaid role as Editor-in-Chief of the International Journal of Diabetes and Technology; and unpaid leadership roles as Central Executive Committee Member of RSSDI and National President of the Diabetes Technology Trust of India. **Arpan Dev** Bhattacharya reports grants or contracts from Abbott Healthcare Pvt Ltd; honoraria for educational events from Abbott; support for attending meetings and/or travel; and participation on a Data Safety Monitoring Board or Advisory Board. **Manash P. Baruah** reports grants or contracts from Abbott Healthcare Pvt Ltd; honoraria for educational events from Abbott; research or educational grants from Abbott, Eli Lilly, Novo Nordisk, Sanofi, Eris, Mankind, Glenmark, Cipla, USV, and Sun Pharma; support for attending meetings and/or travel; and participation on a Data Safety Monitoring Board or Advisory Board. **Ajay Budhwar** reports grants or contracts from Abbott Healthcare Pvt Ltd; honoraria for educational events from Abbott; consultant and speaker fees and research/educational grants from Abbott and Novo Nordisk; support for attending meetings and/or travel; and participation on a Data Safety Monitoring Board or Advisory Board. **Priti Thakor** is an employee of Abbott Healthcare Pvt Ltd and reports grants or contracts from Abbott Healthcare Pvt Ltd and participation on a Data Safety Monitoring Board or Advisory Board. **Sachin Shende** is an employee of Abbott Healthcare Pvt Ltd and reports grants or contracts from Abbott Healthcare Pvt Ltd and participation on a Data Safety Monitoring Board or Advisory Board. **Viswanathan Mohan** reports grants or contracts from Abbott Healthcare Pvt Ltd; consulting fees; honoraria for educational events from Abbott; support for attending meetings and/or travel; participation on a Data Safety Monitoring Board or Advisory Board; and research or educational grants from Abbott, Medtronic, Novo Nordisk, Sanofi, Servier, Boehringer Ingelheim, Eli Lilly, Johnson & Johnson, Lifescan, Roche, MSD, Novartis, Aventis, Bayer, USV, Dr. Reddy’s Laboratories, Sun Pharma, Intas, Lupin, Glenmark, Zydus, IPCA, Torrent, Cipla, Biocon, Primus, Franco Indian, Wockhardt, Emcure, Mankind, Fourrts, Apex, GSK, and Alembic.

## References

[1] Shah V.N., Peters A.L., Umpierrez G.E., Sherr J.L., Akturk H.K., Aleppo G. (2024). Consensus report on glucagon-like peptide-1 receptor agonists as adjunctive treatment for individuals with type 1 diabetes using an automated insulin delivery system. J Diabetes Sci Technol.

[2] (2025). MAGICapp - making GRADE the irresistible choice - guidelines and recommendations.

[3] Neumiller J.J., Bajaj M., Bannuru R.R., McCoy R.G., Pekas E.J., Segal A.R. (2025). Compounded GLP-1 and dual GIP/GLP-1 receptor agonists: a statement from the American diabetes association. Diabetes Care.

[4] Scottish Government (2024). GLP-1 and GLP-1/GIP RA consensus statement for phased implementation.

[5] Gilbert O., Gulati M., Gluckman T.J., Kittleson M.M., Rikhi R., Saseen J.J. (2025). Concise clinical guidance: an ACC expert consensus statement on medical weight management for optimization of cardiovascular health. J Am Coll Cardiol.

[6] Krug I., Dang A.B., Portingale J., Li Y., Won Y.Q. (2025). Beyond weight loss: GLP-1 usage and appetite regulation in the context of eating disorders and psychosocial processes. Nutrients.

[7] Młynarska E., Bojdo K., Bulicz A., Frankenstein H., Gąsior M., Kustosik N. (2025). Obesity as a multifactorial chronic disease: molecular mechanisms, systemic impact, and emerging digital interventions. Curr Issues Mol Biol.

[8] Noronha J.C., Van Gaal L.F., Neeland I.J., Fitch A., Pfeiffer A.F.H., Chiavaroli L. (2025). Optimizing GLP-1 therapies for obesity and diabetes management. Obes Pillars.

[9] Urbina J., Salinas-Ruiz L.E., Valenciano C., Clapp B. (2026). Micronutrient and nutritional deficiencies associated with GLP-1 receptor agonist therapy: a narrative review. Clin Obes.

[10] Christensen S., Robinson K., Thomas S., Williams D.R. (2024). Dietary intake by patients taking GLP-1 and dual GIP/GLP-1 receptor agonists: a narrative review and discussion of research needs. Obes Pillars.

[11] Scott Butsch W., Sulo S., Chang A.T., Kim J.A., Kerr K.W., Williams D.R. (2025). Nutritional deficiencies and muscle loss in adults with type 2 diabetes using GLP-1 receptor agonists: a retrospective observational study. Obes Pillars.

[12] Kim Y.S., Hong K.W., Han K., Park Y.C., Park J.M., Kim K. (2020). Longitudinal observation of muscle mass over 10 Years according to serum calcium levels and calcium intake among Korean adults aged 50 and older: the Korean genome and epidemiology study. Nutrients.

[13] Willoughby D., Hewlings S., Kalman D. (2018). Body composition changes in weight loss: strategies and supplementation for maintaining lean body mass, a brief review. Nutrients.

[14] Caffarelli C., Refaie A.A., Mondillo C., Cavati G., Lora A., Gennari L. (2025). Influence of dietary calcium intake on skeletal health and body composition in an Italian elderly population. Nutrients.

[15] Sachdev M., Misra A. (2023). Heterogeneity of Dietary practices in India: current status and implications for the prevention and control of type 2 diabetes. Eur J Clin Nutr.

[16] Mahajan A., Deshmane A., Muley A. (2025). A comparative study on the consumption patterns of processed food among individuals with and without type 2 diabetes. Int J Publ Health.

[17] Venkatesh U., Sharma A., Ananthan V.A., Subbiah P., Durga R., CSIR Summer Research training team (2021). Micronutrient's deficiency in India: a systematic review and meta-analysis. J Nutr Sci.

[18] Anjana R.M., Sudha V., Abirami K., Gayathri R., Manasa V.S., Deepa M. (2025). Dietary profiles and associated metabolic risk factors in India from the ICMR–INDIAB survey-21. Nat Med.

[19] Lakhani O.J., Shaikh A. (2018). The sweet ‘truth’ of Gujarat – Gujarati diet & lifestyle and diabetogenesis. J Soc Health Diabet.

[20] Dinicola S., Minini M., Unfer V., Verna R., Cucina A., Bizzarri M. (2017). Nutritional and acquired deficiencies in inositol bioavailability. Correlations with metabolic disorders. Int J Mol Sci.

[21] Dixit A.A., Azar K.M.J., Gardner C.D., Palaniappan L.P. (2011). Incorporation of whole, ancient grains into a modern Asian Indian diet: practical strategies to reduce the burden of chronic disease. Nutr Rev.

[22] Mediratta S., Ghosh S., Mathur P. (2023). Intake of ultra-processed food, dietary diversity and the risk of nutritional inadequacy among adults in India. Public Health Nutr.

[23] Lorenzo A.D., Pellegrini M., Gualtieri P., Itani L., Ghoch M.E., Renzo L.D. (2022). The risk of sarcopenia among adults with normal-weight obesity in a nutritional management setting. Nutrients.

[24] Kalra S., Shaikh I.A., Shende S., Kapoor N., Unnikrishnan A.G., Sharma O.P. (2025). An Indian consensus on sarcopenia: epidemiology, etiology, clinical impact, screening, and therapeutic approaches. Int J Gen Med.

[25] Joshi S.R., Bhansali A., Bajaj S., Banzal S.S., Dharmalingam M., Gupta S. (2014). Results from a dietary survey in an Indian T2DM population: a STARCH study. BMJ Open.

[26] Spreckley M., Ruggiero C.F., Brown A. (2026). Bridging the nutrition guidance gap for GLP-1 receptor agonist therapy assisted weight loss: lessons from bariatric surgery. Int J Obes.

[27] Chee W.S.S., Gilcharan Singh H.K., Hamdy O., Mechanick J.I., Lee V.K.M., Barua A. (2017). Structured lifestyle intervention based on a trans-cultural diabetes-specific nutrition algorithm (tDNA) in individuals with type 2 diabetes: a randomized controlled trial. BMJ Open Diabetes Res Care.

[28] Hamdy O., Al Sifri S., Hassanein M., Al Dawish M., Al-Dahash R.A., Alawadi F. (2022). The transcultural diabetes nutrition algorithm: a middle eastern version. Front Nutr.

[29] Gattrell W.T., Logullo P., van Zuuren E.J., Price A., Hughes E.L., Blazey P. (2024). ACCORD (ACcurate COnsensus Reporting Document): a reporting guideline for consensus methods in biomedicine developed via a modified Delphi. PLoS Med.

[30] OCEBM Levels of Evidence Working Group The Oxford Levels of Evidence 2. Oxford Centre for Evidence-Based Medicine. https://www.cebm.ox.ac.uk/resources/levels-of-evidence/ocebm-levels-of-evidence.

[31] Lee S.C., Park Y.H., Singer C.F., Balmaña J., Dent R.A., Tan V.K.M. (2025). Part I: consensus statements and expert recommendations for HER2-negative early breast cancer in the Asia-Pacific region: diagnosis and risk assessment. Front Oncol.

[32] Gorgojo-Martínez J.J., Mezquita-Raya P., Carretero-Gómez J., Castro A., Cebrián-Cuenca A., de Torres-Sánchez A. (2022). Clinical recommendations to manage gastrointestinal adverse events in patients treated with Glp-1 receptor agonists: a multidisciplinary expert consensus. J Clin Med.

[33] Wharton S., Davies M., Dicker D., Lingvay I., Mosenzon O., Rubino D.M. (2022). Managing the gastrointestinal side effects of GLP-1 receptor agonists in obesity: recommendations for clinical practice. Postgrad Med J.

[34] Hagelqvist P.G., Vilsbøll T., Schwarz C.R. (2025). Mechanism and context: making sense of adverse events with GLP-1-based therapy. JCEM Case Rep.

[35] Mozaffarian D., Agarwal M., Aggarwal M., Alexander L., Apovian C.M., Bindlish S. (2025). Nutritional priorities to support GLP-1 therapy for obesity: a joint advisory from the American college of lifestyle medicine, the American society for nutrition, the obesity medicine association, and the obesity society. Am J Clin Nutr.

[36] Kim J.A., Yoo H.J. (2025). Exploring the side effects of GLP-1 receptor agonist: to ensure its optimal positioning. Diabetes Metab.

[37] Jalleh R.J., Talley N.J., Horowitz M., Nauck M.A. (2026). The science of safety: adverse effects of GLP-1 receptor agonists as glucose-lowering and obesity medications. J Clin Investig.

[38] Abdelrahman R.M., Musa T.H., Arbab I.A., Ahmed E.O., Gasmallah S.I., Jalal M. (2026). Adverse effects of GLP-1 receptor agonists: clinical Implications, regulatory perspectives, and future directions. Obes Med.

[39] Winzeler B., Sailer C.O., Coynel D., Zanchi D., Vogt D.R., Urwyler S.A. (2021). A randomized controlled trial of the GLP-1 receptor agonist dulaglutide in primary polydipsia. J Clin Investig.

[40] Fitch A., Gigliotti L., Bays H.E. (2025). Application of nutrition interventions with GLP-1 based therapies: a narrative review of the challenges and solutions. Obes Pillars.

[41] Dong S., Sun C. (2022). Can glucagon-like peptide-1 receptor agonists cause acute kidney injury? An analytical study based on post-marketing approval pharmacovigilance data. Front Endocrinol.

[42] Pomeroy E., Mushrif-Tripathy V., Cole T.J., Wells J.C.K., Stock J.T. (2019). Ancient origins of low lean mass among South Asians and implications for modern type 2 diabetes susceptibility. Sci Rep.

[43] Dulloo A.G., Ramessur V., Hunma S., Joonas N., Ramessur B.N., Schutz Y. (2026). Visceral-to-peripheral adiposity ratio in proneness to sarcopenic obesity: association with low muscle strength, but not low muscle mass, in young women of South Asian descent. Int J Obes (Lond).

[44] Kaur I., Das S., Chandel S., Chandel S. (2025). Possible sarcopenia, sarcopenic obesity phenotypes and their association with diabetes: evidence from LASI wave-1 (2017-18). Diabetes Metab Syndr.

[45] Langer H.T., Joshi A.S., Müller-Werdan U., Norman K. (2026). Causes of sarcopenia and frailty in people taking GLP1RAs. Nat Rev Endocrinol.

[46] Jiao R., Lin C., Cai X., Wang J., Wang Y., Lv F. (2025). Characterizing body composition modifying effects of a glucagon‐like peptide 1 receptor‐based agonist: a meta‐analysis. Diabetes Obes Metab.

[47] Karakasis P., Patoulias D., Fragakis N., Mantzoros C.S. (2025). Effect of glucagon-like peptide-1 receptor agonists and co-agonists on body composition: systematic review and network meta-analysis. Metabolism.

[48] Ren Q., Zhi L., Liu H. (2025). Semaglutide therapy and accelerated sarcopenia in older adults with type 2 diabetes: a 24-month retrospective cohort study. Drug Des Devel Ther.

[49] Alissou M., Demangeat T., Folope V., Van Elslande H., Lelandais H., Blanchemaison J. (2025). Impact of Semaglutide on fat mass, lean mass and muscle function in patients with obesity: the SEMALEAN study. Diabetes Obes Metab.

[50] Neeland I.J., Linge J., Birkenfeld A.L. (2024). Changes in lean body mass with glucagon-like peptide-1-based therapies and mitigation strategies. Diabetes Obes Metab.

[51] Samajdar S., Joshi S. (2025). Semaglutide beyond weight loss: mechanistic insights and functional paradoxes from the SEMALEAN study. Diabetes Obes Metab.

[52] Wilding J.P.H., Batterham R.L., Calanna S., Van Gaal L.F., McGowan B.M., Rosenstock J. (2021). Impact of semaglutide on body composition in adults with overweight or obesity: exploratory analysis of the STEP 1 study. J Endocr Soc.

[53] Caballero-Mateos I., Morales-Portillo C., Aguilera B.G. (2025). Once-Weekly semaglutide improves body composition in Spanish obese adults with type 2 diabetes: a 48-week prospective real-life study. J Clin Med.

[54] McCrimmon R.J., Catarig A.-M., Frias J.P., Lausvig N.L., le Roux C.W., Thielke D. (2020). Effects of once-weekly semaglutide vs once-daily canagliflozin on body composition in type 2 diabetes: a substudy of the SUSTAIN 8 randomised controlled clinical trial. Diabetologia.

[55] Blundell J., Finlayson G., Axelsen M., Flint A., Gibbons C., Kvist T. (2017). Effects of once‐weekly semaglutide on appetite, energy intake, control of eating, food preference and body weight in subjects with obesity. Diabetes Obes Metab.

[56] Look M., Dunn J.P., Kushner R.F., Cao D., Harris C., Gibble T.H. (2025). Body composition changes during weight reduction with tirzepatide in the SURMOUNT‐1 study of adults with obesity or overweight. Diabetes Obes Metab.

[57] Hansen M.S., Wölfel E.M., Jeromdesella S., Møller J.-J.K., Ejersted C., Jørgensen N.R. (2024). Once-weekly semaglutide versus placebo in adults with increased fracture risk: a randomised, double-blinded, two-centre, phase 2 trial. EClinicalMedicine.

[58] Liu Y., Walzer D., Schmitz S., Shukla A.P., Ma X., Chirko D. (2026). Skeletal effect of semaglutide and tirzepatide in patients with increased risk of fractures. J Clin Endocrinol Metab.

[59] Noreña J.A., Pike C.W., Hui G., Motlaghzadeh Y., Sellmeyer D.E., Wu J.Y. (2025). Comparison of fracture risk following semaglutide treatment vs sleeve gastrectomy. AACE Endocrinol Diabetes.

[60] Anastasilakis A.D., Paccou J., Palermo A., Polyzos S.A. (2025). The effects of anti-obesity medications on bone metabolism: a critical appraisal. Diabetes Obes Metab.

[61] Wilding J.P.H., Batterham R.L., Davies M., Van Gaal L.F., Kandler K., Konakli K. (2022). Weight regain and cardiometabolic effects after withdrawal of semaglutide: the STEP 1 trial extension. Diabetes Obes Metab.

[62] Quarenghi M., Capelli S., Galligani G., Giana A., Preatoni G., Turri Quarenghi R. (2025). Weight regain after liraglutide, semaglutide or tirzepatide interruption: a narrative review of randomized studies. J Clin Med.

[63] Budini B., Luo S., Tam M., Stead I., Lee A., Akrami A. (2026). Trajectory of weight regain after cessation of GLP-1 receptor agonists: a systematic review and nonlinear meta-regression. EClinicalMedicine.

[64] Tzang C.C., Wu P.H., Luo C.A., Chen Z.T., Lee Y.T., Huang E.S. (2025). Metabolic rebound after GLP-1 receptor agonist discontinuation: a systematic review and meta-analysis. EClinicalMedicine.

[65] (2026). Less than two years after stopping obesity drugs, weight and health issues return, study finds.

[66] Kolli R.T., Aoutla S., Jyothi N., Mohamed Kalifa M.R.H., Raju A., Cheenikkal Muralidharan K. (2025). Rebound or retention: a meta-analysis of weight regain after the discontinuation of glucagon-like peptide-1 (GLP-1) receptor agonists and other anti-obesity drugs. Cureus.

[67] Rubino D., Abrahamsson N., Davies M., Hesse D., Greenway F.L., Jensen C. (2021). Effect of continued weekly subcutaneous semaglutide vs placebo on weight loss maintenance in adults with overweight or obesity. JAMA.

[68] Horn D.B., Linetzky B., Davies M.J., Laffin L.J., Wang H., Murphy M.A. (2026). Cardiometabolic parameter change by weight regain on tirzepatide withdrawal in adults with obesity. JAMA Intern Med.

[69] ICMR-NIN Expert Committee (2024). https://nin.res.in/dietaryguidelines/pdfjs/locale/DGI_2024.pdf.

[70] Duque C.M., Jackman T. (2024). https://nsuworks.nova.edu/hpd_com_nutrition/18.

[71] Deutz N.E.P., Bauer J.M., Barazzoni R., Biolo G., Boirie Y., Bosy-Westphal A. (2014). Protein intake and exercise for optimal muscle function with aging: recommendations from the ESPEN Expert Group. Clin Nutr.

[72] Kim J.E., O’Connor L.E., Sands L.P., Slebodnik M.B., Campbell W.W. (2016). Effects of dietary protein intake on body composition changes after weight loss in older adults: a systematic review and meta-analysis. Nutr Rev.

[73] Hassapidou M., Vlassopoulos A., Kalliostra M., Govers E., Mulrooney H., Ells L. (2023). European association for the study of obesity position statement on medical nutrition therapy for the management of overweight and obesity in adults developed in collaboration with the European federation of the associations of dietitians. Obes Facts.

[74] Donini L.M., Busetto L., Bischoff S.C., Cederholm T., Ballesteros-Pomar M.D., Batsis J.A. (2022). Definition and diagnostic criteria for sarcopenic obesity: ESPEN and EASO consensus statement. Obes Facts.

[75] Minocha S., Thomas T., Kurpad A.V. (2017). Dietary protein and the health-nutrition-agriculture connection in India. J Nutr.

[76] Zevenbergen H., de Bree A., Zeelenberg M., Laitinen K., van Duijn G., Flöter E. (2009). Foods with a high fat quality are essential for healthy diets. Ann Nutr Metab.

[77] Jacob J., Krishnan V., Antony C., Bhavyasri M., Aruna C., Mishra K. (2024). The nutrition and therapeutic potential of millets: an updated narrative review. Front Nutr.

[78] (2022). RSSDI clinical practice recommendations for the management of type 2 diabetes mellitus 2022. Int J Diabetes Dev Ctries.

[79] Bajaj M., McCoy G.R., Balapattabi K., Bannuru R.R., Bellini N.J., Bennett A.K. (2024). 5. Facilitating positive health behaviors and well-being to improve health outcomes: standards of care in diabetes—2025. Diabetes Care.

[80] Johnson B.V.B., Milstead M., Kreider R., Jones R. (2025). Dietary supplement considerations during glucagon-like Peptide-1 receptor agonist treatment: a narrative review. Obes Pillars.

[81] Mangal D.K., Shaikh N., Tolani H., Gautam D., Pandey A.K., Sonnathi Y. (2025). Burden of micronutrient deficiency among patients with type 2 diabetes: systematic review and meta-analysis. BMJ Nutr Prev Health.

[82] Jayashri R., Venkatesan U., Rohan M., Gokulakrishnan K., Shanthi Rani C.S., Deepa M. (2018). Prevalence of vitamin B12 deficiency in South Indians with different grades of glucose tolerance. Acta Diabetol.

[83] Jayashri R., Venkatesan U., Shanthirani C.S., Deepa M., Anjana R.M., Mohan V. (2020). Prevalence of vitamin D deficiency in urban south Indians with different grades of glucose tolerance. Br J Nutr.

[84] Carr A.C., Vlasiuk E., Zawari M., Lunt H. (2024). Understanding the additional impact of prediabetes and type 2 diabetes mellitus on vitamin C requirements in people living with obesity. Nutr Res.

[85] Mehta N., Akram M., Singh Y.P. (2024). The impact of zinc supplementation on hyperglycemia and complications of type 2 diabetes mellitus. Cureus.

[86] Fatahi M., Aghajani S., Javaheri H., Toqroljerdi M.H.F. (2025). The effect of chromium supplements on insulin resistance: a systematic review and meta-analysis of randomized controlled trials. J Health Med Sci.

[87] Miñambres I., Cuixart G., Gonçalves A., Corcoy R. (2019). Effects of inositol on glucose homeostasis: systematic review and meta-analysis of randomized controlled trials. Clin Nutr.

[88] Tey S.L., Chee W.S.S., Deerochanawong C., Berde Y., Lim L.L., Boonyavarakul A. (2024). Diabetes-specific formula with standard of care improves glycemic control, body composition, and cardiometabolic risk factors in overweight and obese adults with type 2 diabetes: results from a randomized controlled trial. Front Nutr.

[89] Sonkar S.K., Parmar K.S., Ahmad M.K., Sonkar G.K., Gautam M. (2021). An observational study to estimate the level of essential trace elements and its implications in type 2 diabetes mellitus patients. J Fam Med Prim Care.

[90] Astbury N.M., Piernas C., Hartmann-Boyce J., Lapworth S., Aveyard P., Jebb S.A. (2019). A systematic review and meta-analysis of the effectiveness of meal replacements for weight loss. Obes Rev.

[91] Ashley J.M., Herzog H., Clodfelter S., Bovee V., Schrage J., Pritsos C. (2007). Nutrient adequacy during weight loss interventions: a randomized study in women comparing the dietary intake in a meal replacement group with a traditional food group. Nutr J.

[92] Kadouh H., Chedid V., Halawi H., Burton D.D., Clark M.M., Khemani D. (2020). GLP-1 analog modulates appetite, taste preference, gut hormones, and regional body fat stores in adults with obesity. J Clin Endocrinol Metab.

[93] Chiavaroli L., Lee D., Ahmed A., Cheung A., Khan T.A., Blanco S. (2021). Effect of low glycaemic index or load dietary patterns on glycaemic control and cardiometabolic risk factors in diabetes: systematic review and meta-analysis of randomised controlled trials. BMJ.

[94] Lin S., Deed G., Khoo C., Murfet G., Barclay A.W., Maberly G. (2026). Clinical practice guide for integrating diabetes-specific nutritional formulas into diabetes care: evidence review and expert consensus. Diabetology.

[95] Sanz-París A., Matía-Martín P., Martín-Palmero Á., Gómez-Candela C., Camprubi Robles M. (2020). Diabetes-specific formulas high in monounsaturated fatty acids and metabolic outcomes in patients with diabetes or hyperglycaemia. A systematic review and meta-analysis. Clin Nutr.

[96] Chen L.L., Tohit N.M., Ludin A.F.M., Manaf Z.A., Wong A., Kuan K.L. (2025). Efficacy of diabetes-specific partial meal replacement on glycemic and weight control in type 2 diabetes: a randomized controlled trial. Diabetes Obes Metab.

[97] Tan N.C., Joshi S.R., De-Madaria E., Mostafa B.E., Özgirgin O.N., Simoncini T. (2025). Clinicians' view on non-adherence: sharing expert opinion. Front Pharmacol.

[98] Kesari A., Noel J.Y. (2026). http://www.ncbi.nlm.nih.gov/books/NBK580496/.

[99] Cristancho C., Kim D.W., Apovian C.M. (2025). Nutrient-stimulating hormone-based therapies for obesity. Endocrinol Metab Clin North Am.

