# Supplementary material for: Nutritional considerations for glucagon-like peptide-based therapies: An Asian Indian consensus recommendation
Source: Obes Pillars. 2026 Jul 15;19:100296. doi: 10.1016/j.obpill.2026.100296 (PMC13418229; doi:10.1016/j.obpill.2026.100296)
Supplement: Multimedia component 1 [file mmc1.docx]

**Supplementary file**

**Supplementary Table 1:** Oxford Level of Evidence 2011

| **Level** | **Epidemiology** | **Diagnosis** | **Prognosis** | **Treatment benefits** | **Treatment harms** | **Screening** |
| --- | --- | --- | --- | --- | --- | --- |
| **Level 1** | Systematic review of high-quality population-based surveys or censuses | Systematic review of high-quality cross-sectional studies with blinding and reference standard | Systematic review of inception cohort studies | Systematic review of RCTs or n-of-1 trials | Systematic review of RCTs or large observational studies | Systematic review of RCTs |
| **Level 2** | Individual high-quality population-based survey | Individual high-quality cross-sectional study with reference standard | Individual inception cohort study | Individual RCT or observational study with large effect | Individual RCT or well-designed observational study | Individual RCT |
| **Level 3** | Non-random or regional surveys | Non-consecutive studies or studies with methodological limitations | Retrospective cohort study or control arm of RCT | Non-randomized controlled cohort or follow-up study | Observational cohort with sufficient data | Non-randomized cohort or follow-up study |
| **Level 4** | Case series | Case–control studies or poor reference standard | Case series or poor-quality cohort studies | Case series or case–control studies | Case series, case–control studies, or historical controls | Case series or case–control studies |
| **Level 5** | Mechanism-based reasoning or expert opinion | Mechanism-based reasoning or expert opinion | Mechanism-based reasoning or expert opinion | Mechanism-based reasoning or expert opinion | Mechanism-based reasoning or expert opinion | Mechanism-based reasoning or expert opinion |

**Supplementary Table 2:**Quality of evidence and definitions

| **Code** | **Quality of evidence** | **Definition** |
| --- | --- | --- |
| A | High | Further research is very unlikely to change our confidence in the estimate of effect.   - Several high‐quality studies with consistent results - In special cases—one large, high‐quality multicenter trial |
| B | Moderate | Further research is likely to have an important impact on our confidence in the estimate of effect and may change the estimate.   - One high‐quality study - Several studies with some limitations |
| C | Low | Further research is very likely to have an important impact on our confidence in the estimate of effect and is likely to change the estimate.   - One or more studies with severe limitations |
| D | Very low | Any estimate of the effect is very uncertain.   - Expert opinion - No direct research evidence - One or more studies with very severe limitations |

**Supplementary Table 3:**Level of consensus: definitions

| **Level of consensus** | **Definition** |
| --- | --- |
| High | When ≥75% of participants agree or disagree with a statement. |
| Moderate | When 55%–74% of participants agree or disagree with a statement. |
| Low | When <55% of participants agree or disagree with a statement. |
